# In silico Drug Repurposing to combat COVID-19 based on Pharmacogenomics of Patient Transcriptomic Data

**DOI:** 10.21203/rs.3.rs-39128/v1

**Published:** 2020-06-30

**Authors:** Shaoli Das, Kevin Camphausen, Uma Shankavaram

**Affiliations:** National Institutes of Health, National Cancer Institute; National Institutes of Health, National Cancer Institute; National Institutes of Health, National Cancer Institute

**Keywords:** SARS-CoV-2, COVID-19, drug repurposing, patient transcriptomics, pharmacogenomics, insilico

## Abstract

The ongoing global pandemic of coronavirus disease 2019 (COVID-19) continues to affect a growing number of populations in different parts of the world. In the current situation, drug repurposing is a viable strategy to combat COVID-19. The drugs targeting the host receptors that interact with SARS-CoV-2 are possible candidates. However, assessment of their effectiveness in COVID-19 patients is necessary before prioritizing them for further study. We attempted to shortlist the candidate drugs using an in-silico approach. First, we analysed two published transcriptomic data sets of COVID-19- and SARS-infected patients compared to healthy individuals to find the key pathways altered after infection. Then, using publicly available drug perturbational data sets in human cell lines from the Broad Institute Connectivity Map (CMAP), we assessed the effects of the approved drugs on the altered pathways. We also used the available pharmacogenomic data sets from the Genomics of Drug Sensitivity in Cancer (GDSC) portal to assess the effects of the altered pathways on resistance or sensitivity to the drugs in human cell lines. Our analysis identified many candidate drugs, some of which are already being investigated for treatment of COVID-19 and can serve as a basis for prioritizing additional viable candidate drugs for COVID-19.

## Introduction

In December 2019, outbreak of a new virus causing severe respiratory disease was reported in Wuhan, China. The disease was attributed to infection by a novel member of the coronavirus family, named SARS-CoV-2^1^. In contrast with the two other highly pathogenic coronaviruses, SARS-CoV (outbreak in 2002) and MERS-CoV (outbreak in 2012), the novel virus SARS-CoV-2 had a lower fatality rate, but it could spread from person to person more efficiently^2^. The outbreak spread worldwide^3^, and by March 2020, the World Health Organization declared the disease, by then named coronavirus disease 2019 (COVID-19), a global pandemic. COVID-19 is currently a leading public health concern in countries worldwide as the hospitalization rate and death toll increase daily (https://www.who.int/emergencies/diseases/novel-coronavirus-2019). Apart from mitigation efforts, there is an urgent need for effective treatments for patients showing severe symptoms. After Chinese researchers published the first sequence of SARS-CoV-2 in January^4^, many groups worldwide began working to develop an effective vaccine. Some of the candidates are undergoing clinical trials, among them two promising candidates from Oxford University and Moderna, Inc., that are entering Phase II^5^. However, clinical approval of the vaccines will take some time; consequently, the best solution in the interim is to look for drugs that have already been approved for humans and that may be repurposed for COVID-19 treatment. The U.S. Food and Drug Administration recently approved remdesivir, an antiviral drug targeting the viral RNA-dependent RNA polymerase, for COVID-19 treatment after a clinical trial run by the National Institute of Allergy and Infectious Diseases found it to reduce the time to recovery in treated patients compared to the control group^6^. However, the drug did not show significant improvement in patient survival. Thus, there is still a persistent need to search for alternative candidate drugs that can be used as single or as combination therapy to benefit patients with severe illness.

Researchers are also exploring targeting of the host receptors of SARS-CoV-2 as another potential solution. Inhibitors of the two main receptors for SARS-CoV-2/SARS-CoV-1 entry in human cells, ACE2 and TMPRSS2, are being tested as potential treatment options^7^. Other host interactors have been curated from previously published literature in a recent study^8^, and a list of 332 high-confidence interactions between SARS-CoV-2 and human proteins was recently published by Gordon et al., who used affinity purification mass spectrometry on a human cell line expressing the viral proteins^9^.

**Figure 1:**
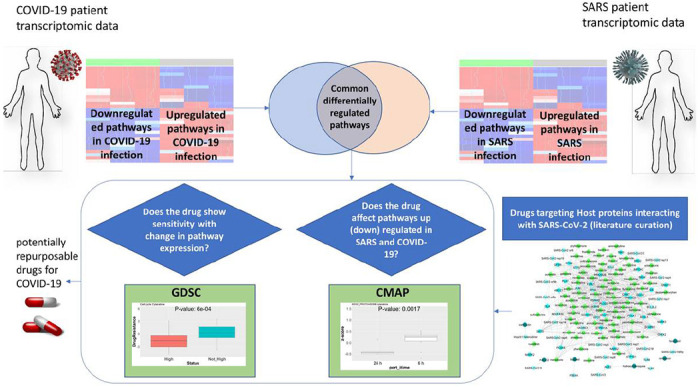
Graphical description of our approach to systematically assess candidate drugs that can be re-purposed for Covid-19 (or SARS) treatment, based on the transcriptomic changes of infected patients. We combined recently published COVID-19-infected patient transcriptomic data and previous SARS-infected patient transcriptomic data, to find key pathway alterations in infected patients. On the other hand, we collected the drugs targeting these SARS-CoV-2/SARS-CoV-1-interacting proteins from DrugBank and DGIdb to assess their effect in COVID-19- and SARS-infected patients, in-silico. Next, using the available drug perturbational data sets from the Broad Institute CMAP project, we assessed how the candidate drugs targeting SARS-CoV-2-interacting proteins affect the pathways that are altered after COVID-19 or SARS infection in a time-dependent manner. Using another available pharmacogenomic data resource, Genomics of Drug Sensitivity in Cancer (GDSC), we assessed whether the altered pathways affect sensitivity or resistance to our candidate drugs in human cell lines. Finally, we present a list of potential drugs for COVID-19 treatment that affect the COVID-19-altered pathways and incur less resistance in the presence of alteration in the pathways.

We collected the drugs targeting these SARS-CoV-2/SARS-CoV-1-interacting proteins from DrugBank^10^ and the Drug Gene Interaction Database (DGIdb)^11^ and assessed their effect in COVID-19- and SARS-infected patients in silico using published patient transcriptomic data and drug perturbational data sets. By combining recently published COVID-19-infected patient transcriptomic data^12^ and previous SARS-infected patient transcriptomic data^13^, we found key pathways that are altered in infected patients compared to healthy individuals. The common altered pathways were related to androgen biosynthesis, steroid biosynthesis, gluconeogenesis, regulation of mitosis, the proteasome, and inflammatory pathways. Next, using the available drug perturbational data sets from the Broad Institute Connectivity map (CMAP project^14^, we assessed how the candidate drugs targeting SARS-CoV-2-interacting proteins affect the pathways that are altered after COVID-19 or SARS infection in a time-dependent manner. Using another available pharmacogenomic data resource, Genomics of Drug Sensitivity in Cancer (GDSC)^15^, we assessed whether the altered pathways affect sensitivity or resistance to our candidate drugs in human cell lines. Finally, we present a list of potential drugs for COVID-19 treatment that affect the COVID-19-altered pathways and incur less resistance in the presence of alteration in the pathways. Figure 1 summarizes our approach.

## Results

### Analysis of transcriptomic data of COVID-19- and SARS-infected patients revealed common altered pathways

**Figure 2:**
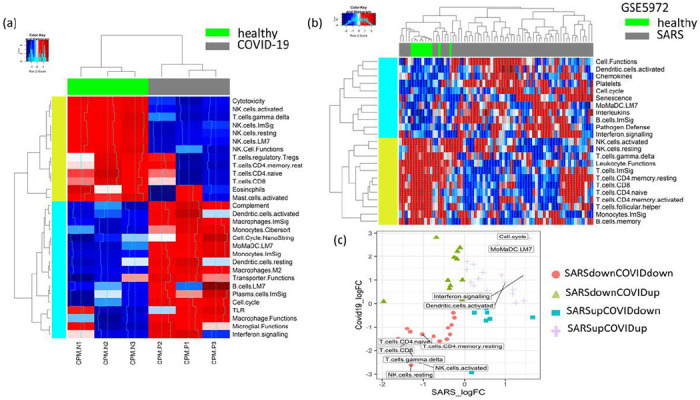
(a) The heat map compares the immunologic signatures (ssGSEA) between the PBMCs of COVID-19 and healthy patients. COVID-19 patients have increased enrichment of MoMaDC; toll-like receptors (TLR); plasma cells; B cells; cell cycles; and interferon and complement functions, while the NK-cell and T-cell functions have decreased enrichment. (b) The heat map compares the PBMCs of SARS and healthy patients. SARS patients have increased enrichment of MoMaDC, B cells, cell cycles, chemokines, and interferon and interleukins functions, while the natural killer (NK)-cell and T-cell functions have decreased enrichment. (c) The scatter plot shows the log fold changes of differentially enriched immune signatures in SARS vs. healthy and COVID-19 vs. healthy patients. The common immune signatures highly enriched in both SARS- and COVID-19-infected patients (top right panel, purple) are MoMaDC functions, interferon signaling, and cell cycle, while the common immune signatures with low enrichment (bottom left panel, pink) are NK- and T-cell functions.

**Figure 3:**
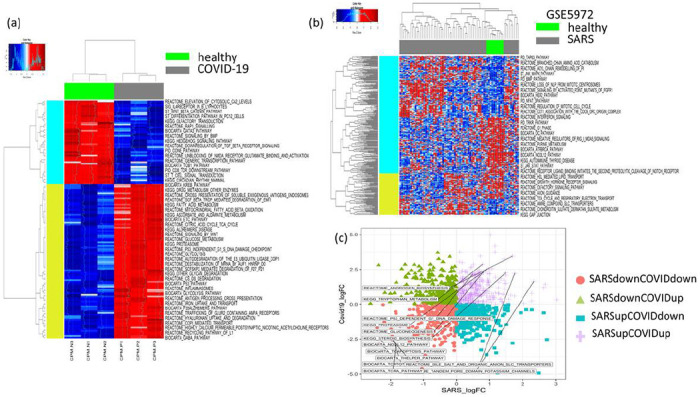
(a) The heat map compares the MSigDB pathway signatures (ssGSEA) between the PBMCs of COVID-19 and healthy patients. COVID-19 patients have increased enrichment of glucose metabolism, glycolysis, Wnt signaling, Krebs cycle, proteasomes, p53 pathway, while the T-cell receptor (TCR), T helper, cytotoxic T lymphocyte (CTL) pathways have decreased enrichment. (b) In the heat map comparing the PBMCs of SARS and healthy patients, SARS patients have increased enrichment of cell cycle and tricarboxylic acid (TCA) cycle functions, while the TCR and CTL functions have decreased enrichment. (c) The scatter plot shows the log fold changes of differentially enriched pathway signatures in SARS vs. healthy and COVID-19 vs. healthy patients. The common pathway signatures highly enriched in both SARS- and COVID-19-infected patients (top right panel, purple) are androgen receptor, solute carrier transport, and potassium channel pathways, while the common immune signatures with low enrichment (bottom left panel, pink) are TCR, T-cell apoptosis, T helper, and CTL pathways.

To get an understanding of the transcriptomic alterations in COVID-19-infected patients, we analysed a published RNA sequencing (RNA-Seq) data set of peripheral blood mononuclear cells (PBMC) of three COVID-19 patients from China compared to three healthy individuals^12^. As there were not enough patient transcriptomic data available for COVID-19-infected people, we searched the literature for transcriptomic data sets of SARS-infected patients. We included a microarray-based transcriptomic data set of PBMCs of 40 SARS-infected and 10 heathy individuals from a paper published in 2007^13^. For both of these transcriptomic data sets, we performed single-sample Gene Set Enrichment Analysis (ssGSEA) for a set of curated pathways collected from the Broad Institute Molecular Signatures Database (MSigDB) and a set of immune function pathways collected from published resources^16,18^. While checking the differential enrichment of immune functions between COVID-19 vs. healthy as well as SARS vs. healthy patients, we found that the macrophages, monocytes, dendritic cells (MoMaDC), B cells, pathogen defence, and interferon signalling were the common immune functions with high enrichment in both COVID-19- and SARS-infected patients compared to healthy patients (Figure 2). On the other hand, among other pathways, androgen biosynthesis, steroid biosynthesis, potassium channel, proteasomes, glycolysis, gluconeogenesis, tryptophan metabolism, and p53-dependent DNA damage response were the common functions that were highly enriched in both COVID-19- and SARS-infected patients compared to healthy patients (Figure 3). Some of the common pathways with less enrichment in the COVID-19- and SARS-infected patients are NK-cell- and T-cell-mediated responses (Figure 2c, 3c).

### Assessing the effect of potential drug candidates on the pathways altered in COVID-19 and SARS infection

**Figure 4:**
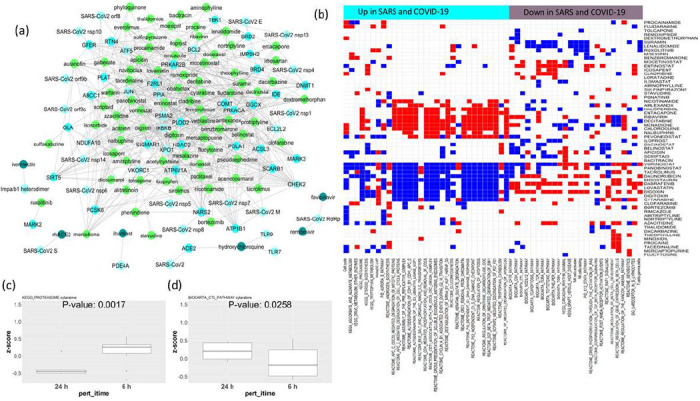
(a) The network of drugs targeting the host (human) proteins interacting with SARS-Cov-1 and SARS-Cov-2. The light green nodes are the drugs that downregulate the expression of the host genes that interact with SARS-Cov-1 or SARS-Cov-2. The interactions of SARS-Cov-2 with host proteins are collected from Gordon et al.9; the drugs targeting the host proteins are collected from DrugBank and DGIdb10,11; and the drug-induced changes in gene expression (time- and dose-dependent) in cell lines are collected from CMAP14. The dark green nodes are currently in clinical trials for COVID-19 (collected from literature). (b) The map shows the drugs from Figure 3a that significantly affect the pathways upregulated (cyan column-side colors) or downregulated (purple column-side colors) in SARS and COVID-19 infection. Blue cells indicate the pathway gets significantly downregulated by the drug (p < 0.05), while red indicates the pathway gets significantly upregulated by the drug in a time-dependent manner (measured by differences in pathway ssGSEA scores at 24 hours vs. 6 hours of drug treatment from CMAP). (c-d) The boxplots show the differences in ssGSEA scores for the pathways KEGG_Proteasomes (high in SARS and COVID-19 infection) and BIOCARTA_CTL_pathway (low in SARS and COVID-19 infection) at 6 hours vs. 24 hours of treatment with the drug cytarabine. Significant downregulation of KEGG_Proteasomes and significant upregulation of BIOCARTA_CTL_pathway are seen as an effect of cytarabine at 24 hours compared to 6 hours (cell line drug treatment data from CMAP).

In an attempt at finding already approved drugs that could be repurposed for COVID-19 treatment, we explored the list of all human proteins interacting with SARS-CoV-2 that were collected from the affinity capture mass-spectrometry study by Gordon et al.^9^ and the list of SARS-CoV-1-interacting proteins curated from previous literature^8^. We collected the list of drugs targeting these host proteins from DrugBank and DGIdb databases^10–11^. Additionally, we procured the list of drugs currently in trial for COVID-19 treatment from literature (https://www.excelra.com/COVID-19-drug-repurposing-database/data). The resulting list comprised 289 potential drugs for COVID-19. Using the drug perturbational data sets from CMAP^14^, we found the drugs that affected the gene expression of these targets (differences in gene expression at 24 hours vs. 6 hours of drug treatment). We mapped 221 drugs to the drug perturbational data set from CMAP. The network in Figure 4a shows the drugs and SARS-CoV targets, which get significantly downregulated at 24 hours compared to six hours of the respective drug treatments (76 drugs) along with the drugs in clinical trial for COVID-19 treatment (Figure 4a, Supplementary Dataset 1 shows the list of drugs and their targets). Many of these drugs have been already reported as potential treatments for COVID-19 and/or SARS (Table 1), including sirolimus, apicidin, mercaptopurine, midostaurin, camostat, metformin, fedratinib, ribavirin, entacapone, azacitidine, and chloroquine. Next, we sought to check if the drugs are effective in the context of transcriptomic changes in COVID-19 or SARS patients after infection. We checked the effect of these drugs on the perturbation of the pathways upregulated or downregulated after SARS and COVID-19 infection (Figure 4b). In CMAR gene expression is profiled in cell lines treated with drugs at different dose/time points, and we see how drugs change the expression of a given set of genes. To get a pathway-based estimation instead of individual genes, we calculated ssGSEA scores for the differentially enriched pathways in COVID-19 or SARS infection for all drug-treated cell lines at different dose/time points. Then the ssGSEA scores of a pathway were compared between two time points (24 hours vs. 6 hours) in cells treated with a certain drug using student’s t-test. Among all drugs targeting SARS-CoV-2-interacting proteins, 60 drugs altered the COVID-19/SARS differentially enriched pathways in a time-dependent manner. In Figure 4b (see Supplementary Dataset 2 for related data), we show drugs that 1) significantly downregulate the pathways that get upregulated after COVID-19/SARS infection (blue cells on the left panel), 2) upregulate the pathways that get upregulated after COVID-19/SARS infection (red cells on the left panel), 3) upregulate the pathways that get downregulated after COVID-19/SARS infection (red cells on the right panel), and 4) downregulate the pathways that get downregulated after COVID-19/SARS infection (blue cells on the right panel). Among these drugs, vorinostat, panobinostat, tacrolimus, lovastatin, midostaurin, daunorubicin, cytarabine, digitoxin, digoxin, mercaptopurine, bortezomib, azacitidine, and clorafarabine can suppress the pathways that were upregulated due to COVID-19/SARS infection (p-value < 0.05, t-test with alternative hypothesis = less), while they can also boost the pathways that were downregulated due to COVID-19/SARS infection (p-value < 0.05, t-test with alternative hypothesis = greater). As examples, Figure 4c and 4d show the effect of the drug cytarabine on the proteasome (upregulated in COVID-19/SARS infection) and CTL (downregulated in COVID-19/SARS infection) pathways at 24 hours vs. 6 hours of drug treatment. In contrast, another class of drugs, including entacapone, ribavirin, decitabine, menadione, chloroquine, dacinostat, and belinostat, significantly upregulated the pathways upregulated in COVID-19 orSARS infection.

### Assessing the effect of potential drug candidates on the pathways altered in COVID-19 and SARS infection

**Figure 5:**
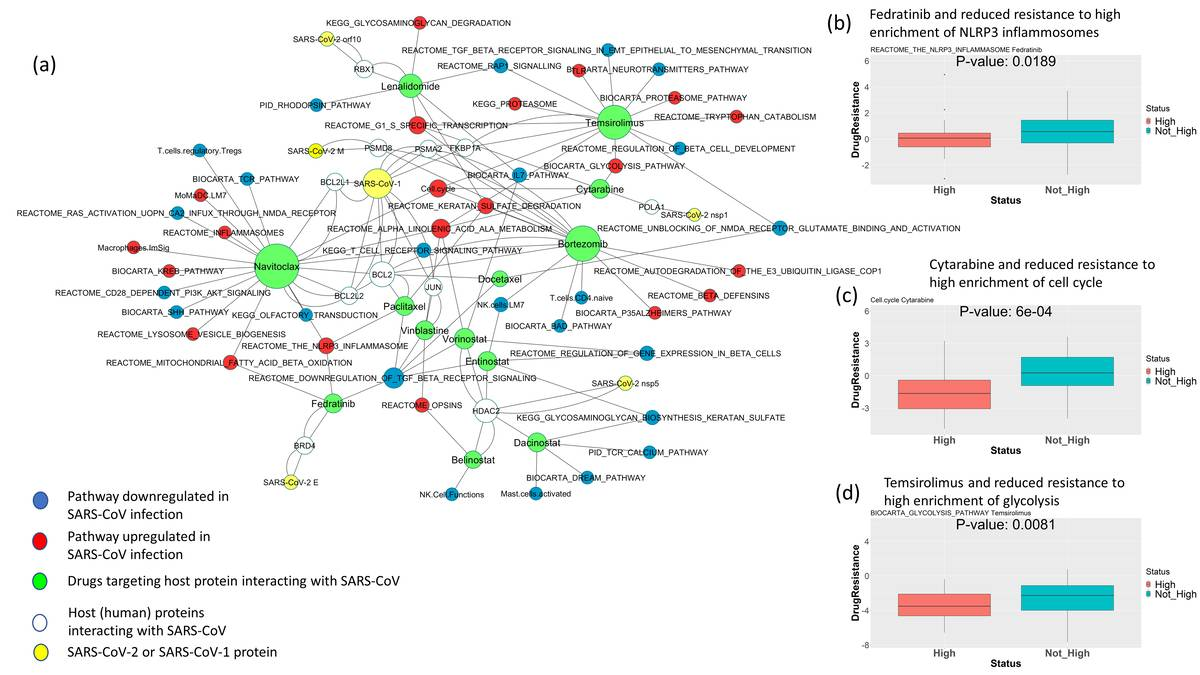
(a) The network shows the repurposable drugs associated with reduced resistance to the pathways upregulated or downregulated in SARS and COVID-19 infection. Interactions of SARS-Cov-1 or SARS-Cov-2 with host proteins are collected from Gordon et al.; the drugs targeting the host proteins are collected from DrugBank and DGIdb10,11; and the pathways upregulated or downregulated in COVID-19 infection that makes cell lines less resistant to the drugs targeting SARS-Cov-2-interacting proteins are calculated using cell line drug screening and cell line transcriptomic data from GDSC. (b-d) The boxplots show the differences in resistance (IC50) to the drugs (b) fedratinib, (c) cytarabine, and (d) temsirolimus in cell lines of hematopoietic or lymphoid origin from GDSC when (b) NLRP3 inflammosomes, (c) cell cycle, and (d) glycolysis are highly enriched (scaled ssGSEA score > 2) vs. not enriched. For pathways downregulated in COVID-19-infected patients, the cell line drug resistance is compared between cells where the pathway has low enrichment (scaled ssGSEA < −2) vs. other cells.

Drug treatment sensitivity or resistance also depends on the genomic and transcriptomic features of tissues. The upregulated or downregulated pathway signatures of virus-infected patients can impact the sensitivity or resistance to the drugs targeting SARS-Cov-2-interacting proteins. Considering this, we sought to check whether the pathways that get altered after SARS or COVID-19 infection have any effect on drug resistance. Drug sensitivity is calculated from GDSC^15^. If the upregulated (or downregulated) pathways are associated with reduced half maximal inhibitory concentration (IC50) of a drug targeting a SARS-CoV-interacting protein, then we can infer that the pathway alteration makes the cell lines less resistant to drug treatment. Figure 5a (see Supplementary Dataset 3 for related data) shows the network of drug and pathway relations from this analysis, where upregulation (or downregulation) of these pathways is associated with reduced resistance to the drugs in GDSC cell lines. The network includes drugs targeting SARS-CoV-interacting proteins and common pathways altered (upregulated or downregulated) in COVID-19/SARS infection. Figure 5b illustrates that the high enrichment of NLRP3 inflammosomes (upregulated in COVID-19-and/or SARS-infected patients) is associated with reduced resistance (low IC50) to the drug fedratinib (calculated from GDSC data). Similarly, Figure 5c and 5d show reduced resistance to the drugs cytarabine and temsirolimus in GDSC cell lines as affected by high enrichment of the pathways’ cell cycle and glycolysis.

## Discussion

Considering the time needed for developing a functional vaccine, the feasible way to fight the COVID-19 pandemic is repurposing drugs already approved for clinical use. We tried to address this issue by identifying drugs that target potential host proteins interacting with SARS-CoV-2 and predicting their effect on the patient transcriptomic signatures of COVID-19- and/or SARS-infected patients. We analyzed COVID-19- and SARS-infected patients’ and healthy individuals’ transcriptomic data sets^12,13^ to identify the pathways commonly altered due to infection with these viruses. Thereafter, combining the potential human interactome of SARS-CoV-2 from a recently published study^9^ and SARS-CoV-1-interacting proteins curated in another publication^8^ with drug target databases^10,11^, drug perturbational data sets^14^, and drug sensitivity screening data sets^15^, we propose a map of the drugs that can be effective in COVID-19 treatment. Our proposed framework for assessing drug efficacy is based on two approaches: 1) whether the drugs can regulate the pathways that get altered due to COVID-19/SARS infection and 2) whether the sensitivity or resistance to the drugs depends on the alteration of these pathways.

We acknowledge that there are some limitations to our study. First, we found only one publicly available data set for transcriptomic signatures of COVID-19-infected patients, and that study only included three patients and three healthy individuals^12^. Since it would be difficult to draw meaningful conclusions on the patient transcriptomic changes from so few patient samples, we included a previously published data set of SARS-infected patient transcriptomes consisting of 40 SARS-infected patients and 10 healthy individuals^13^. We reasoned that the viruses SARS-CoV-1 (causing SARS) and SARS-CoV-2 (causing COVID-19) share many similarities, and the symptoms of the infected patients are also similar, though SARS infection causes more severe symptoms and a higher fatality rate^19^. Another limitation of our study is the availability of drug perturbational and drug sensitivity screening data in human cell lines. Many of the drugs targeting the potential SARS-CoV-2- or SARS-CoV-1-interacting proteins did not have drug perturbational data available in human cell lines from CMAR and only a few drugs have the drug sensitivity screening data available from GDSC, which is limited to drugs commonly used in cancer treatment. Without the available drug perturbational data, we could not assess the effects of antiviral drugs like remdesivir, favipiravir, and oseltamivir in the human transcriptome and were limited to drugs targeting the potential human receptors of the viruses.

Our analysis of COVID-19- and SARS-infected patient transcriptomic signatures identified the common pathways upregulated or downregulated after infection with these viruses. Among the common pathways, androgen biosynthesis, steroid biosynthesis, proteasome, tryptophan metabolism, and mitotic cell cycle-related pathways were upregulated due to infection, while TCR, Interleukin 2, TGF-beta receptor signaling, and beta-cell development pathways were downregulated. Among the immune pathways, MoMaDC, B cells, and interferon responses were elevated in patients with COVID-19 and SARS infection, while T-cell and NK cell functions were downregulated. Many of these pathways have been previously implicated in viral infections. Androgens have already been linked to the severity of COVID-19 as possible mechanisms driving disease outcomes^20^. Steroid, proteasome, and tryptophan metabolisms are linked to viral infections and subsequent host immune response^21,23^. Moreover, monocyte and macrophage activation has been linked to the hyperinflammatory phenotypes in SARS-CoV-2 infection that have adverse clinical outcomes^24^. By analysing the drug perturbational data sets from CMAR we identified drugs that can upregulate or down regulate the pathways that get altered due to COVID-19/SARS infection. As at this point, it’s unclear which pathways will be clinically beneficial for COVID-19 treatment when inhibited or elevated. We provided the full map of all drugs that 1) downregulate the pathways that get upregulated due to COVID-19/SARS infection, 2) upregulate the pathways that get upregulated due to COVID-19/SARS infection, 3) upregulate the pathways that get downregulated due to COVID-19/SARS infection, and 4) downregulate the pathways that get downregulated due to COVID-19/SARS infection. Some drugs that could downregulate the androgen biosynthesis pathway (upregulated due to COVID-19/SARS infection), which is linked to severe outcomes, are ponatinib, amlexanox, lenalidomide, menadione, and decitabine. The drugs vorinostat, panobinostat, tacrolimus, lovastatin, midostaurin, daunorubicin, cytarabine, digitoxin, digoxin, mercaptopurine, bortezomib, azacitidine, and clofarabine could downregulate the mitotic cell cycle-related pathways and proteasome activity. The mitotic cell-cycle arrest and uniquitin-proteasome pathway systems play an important role in viral replication for coronaviruses^22,25^. Drugs that could downregulate the MoMaDC cell pathway potentially linked to severe symptoms in COVID-19 patients^24^ are menadione, decitabine, amlexanox, mercaptopurine, nicotinamide, and pevonedistat. The drugs that could upregulate the CTL or Interleukin 2 pathways, which are important for antiviral immune responses, are: icosapent, lovastatin, azacitidine, decitabine, vorinostat, entinostat, digoxin, ponatinib, apicidin, daunorubicin, and metformin. Many of these drugs have been recently reported as possible treatment options for COVID-19, including ponatinib, mercaptopurine, azacitidine, apicidin, daunorubicin, and metformin^9^.

Considering that host transcriptomic features can affect the sensitivity or resistance to drugs^15^, we checked whether the pathways that get altered after COVID-19/SARS infection can reduce the resistance to the drugs under consideration using GDSC cell-line drug sensitivity screening data. We were limited to drugs approved for cancer treatment that have been screened in human cancer cell lines, so we could not test all potential drugs for COVID-19. However, among the drugs we tested, we found that there was reduced drug resistance (increased sensitivity) related to the pathway alteration signature due to COVID-19/SARS infection with the drugs navitoclax, bortezomib, fedratinib, lenalidomide, cytarabine, temsirolimus, paclitaxel, docetaxel, vorinostat, belinostat, entinostat, dacinostat, and vinblastine. Among these drugs, fedratinib is a potential treatment option for COVID-19^26^. Fedratinib is a semi-selective janus kinase inhibitor with anti-inflammatory properties that can be beneficial for treatment of severe COVID-19 patients with hyperinflammatory symptoms that may lead to lung damage and death. Among the other cancer drugs, the histone deacetylase inhibitors vorinostat, belinostat, entinostat, and dacinostat have anti-inflammatory properties, and the drug lenalidomide has immunomodulatory properties that may have treatment benefits in COVID-19 patients with severe symptoms, possibly when treated with antiviral agents. The chemotherapeutic drugs paclitaxel, docetaxel, and cytarabine may also have potential antiviral properties^27,28^; however, their suitability for treating virus infection is not well established, and therefore, these drugs should be carefully examined before being considered as treatment options.

Our computational study provides an assessment of the potential effects of possible repurposable drugs targeting SARS-CoV-2- and SARS-CoV-1-interacting proteins in the context of transcriptomic signatures of COVID-19-or SARS-infected patients. Because we have limited understanding of the virus pathogenesis and the safety of targeting specific pathways for treatment of this disease, we have included a complete map of the drug-pathway relationships that can be important in the context of COVID-19 infection and treatment. Due to the urgency of identifying effective drugs for treatment of COVID-19 patients, many groups have been working on repurposing existing drugs that can be used for this disease^8^. Our study may be the first that links potentially repurposable drugs to the pathway signatures of infected patients. The drugs we presented in this study as potentially repurposable for COVID-19 treatment should be clinically tested for efficacy before use.

## Methods

### Data sets and pre-processing

The RNA-Seq transcriptomic data of the PBMCs from three COVID-19 infected patients and three healthy individuals were obtained from the study by Xiong et al.^12^. The microarray transcriptomic data of the PBMCs from 40 SARS-infected and 10 healthy patients were obtained from the study by Cameron et al.^13^. The RNA-Seq data from the COVID-19 patients and healthy controls was normalized to fractions per kilobase per million (FPKM). The microarray data from SARS patients and healthy controls was normalized with locally weighted scatterplot smoothing (LOWESS). Transcriptomic data from both COVID-19 and SARS studies were log2-transformed before further processing.

The list of human proteins interacting with SARS-CoV-2 was collected from the study by Gordon et al.^9^. In addition, the human proteins interacting with SARS-CoV are collected from the article by Zhou et al.^8^. The drugs targeting the SARS-CoV-2-interacting proteins were collected from DrugBankand DGIdb^10–11^. The drug perturbational data set containing dose- and time-dependent transcriptomic profiles upon drug treatments in human cell lines was collected from CMAP^14^. The drug-screening data measuring IC50 upon drug treatments in human cell lines and cell-line transcriptomic profiles are collected from GDSC^15^.

### Computational analysis and metadata

The general pathway gene sets were obtained from the C2 collection of MSigDB^29^. We curated immunologic signatures specific to immune functions from four published resources: the LM22 immune infiltration signature^16^, the LM7 immune infiltration signature^17^, the ImSig signature of solid tumor immune infiltration^18^, and the NanoString immune signature panel (https://www.nanostring.com). The combined 3,129 curated gene sets and 61 immune gene sets were used for a single-sample gene-set enrichment analysis on the RNA-seq and microarray transcriptomic tumor data sets for COVID-19- and SARS-infected patient transcriptomes as well as for cell-line transcriptomic data for the CMAP drug perturbational studies and GDSC data sets. R-package gene-set variation analysis was used^30^. Differential enrichments of pathways between COVID-19- and SARS-infected patients vs. healthy controls were calculated with limma R package using ssGSEA scores. The pathways with adjusted p-value < 0.05 are identified as differentially enriched.

A one-sided student’s t-test was used for calculating the p-value of differences in pathway ssGSEA scores between time-dependent drug treatment responses (from CMAP) at 24 hours vs. 6 hours. For pathways that are differentially regulated due to COVID-19/SARS infection, we checked whether a drug can significantly downregulate the pathway at 24 hours compared to 6 hours of transfection in human cell lines from CMAP by performing t-tests on the pathway ssGSEA scores between these two time points using an alternative hypothesis equal to less. Similarly, to check whether a drug can significantly upregulate the pathway at 24 hours compared to 6 hours of transfection, we performed t-tests on the pathway ssGSEA scores between these two time points using an alternative hypothesis equal to greater.

The pathway-dependent drug sensitivity/resistance for each pathway and drug combination from GDSC is calculated using the following approach. First, ssGSEA scores for the pathways are calculated for the GDSC cell lines. Then, the cell lines are stratified into two sets based on the ssGSEA scores of the pathways. For upregulated pathways, cell lines with pathway ssGSEA z-score > 2 have high pathway enrichment, and for downregulated pathways, cell lines with pathway ssGSEA z-score < −2 have low pathway enrichment. The difference between drug IC50 in GDSC cell lines with high ssGSEA scores (z-score > 2) for COVID-19/SARS upregulated pathways and all other GDSC cell lines was calculated using a one-sided t-test with an alternative hypothesis equal to greater. The difference between drug IC50 in GDSC cell lines with low ssGSEA scores (z-score < −2) for COVID-19/SARS downregulated pathways and all other GDSC cell lines was calculated using a one-sided t-test with an alternative hypothesis equal to less.

## Supplementary Material

Supplement Supplementary Dataset 1. Data related to Figure 4a. The list of potential drugs targeting SARS-CoV-2 or SARS-CoV-1 interacting proteins.

Supplement Supplementary Dataset 2. Data related to Figure 4b. Drugs that significantly alter the pathways that get affected after COVID-19/SARS infection.

Supplement Supplementary Dataset 3. Data related to Figure 5a. Drug and pathway relations, where upregulation (or downregulation) of the pathway is associated with reduced resistance to the drugs in GDSC cell lines.

## Figures and Tables

**Table 1.** Literature evidence of drugs that can be repurposed to target SARS-CoV-2-interacting proteins.

Drug	Targets interacting with SARS-CoV-2	Literature/clinical trial evidence
sirolimus	PSMA2,ACSL3,SCARB1,PLOD2,POLA1,XPO1,COMT,BCL2L2,IKBKB,CHEK2,PPIA,GLA,SIGMAR1,IDE	clinical trial: NCT04341675
irbesartan	BCL2,GFER,IKBKB,SIRT5,NARS2	PMID: 32129518
ribavirin	ATP6V1A,PLOD2,BCL2,GGCX,BCL2L2,IMPDH2	PMID: 32227493
apicidin	PSMA2,DNMT1,SCARB1,PLAT,PLOD2,ATF5,PCSK6,COMT,CHEK2,RTN4,SIGMAR1,HDAC2	PMID: 32353859
azacitidine	PSMA2,JUN,SCARB1,ABCC1,PRKAR2B,PCSK6,COMT,CHEK2,F2RL1,BRD2	PMID: 32353859
entacapone	JUN,DNMT1,PRKACA,ABCC1,COMT,F2RL1,TBK1	PMID: 32353859
mercaptopurine	DNMT1,SCARB1,HDAC2,PRKAR2B,XPO1,COMT,PPIA,IDE,NARS2	PMID: 18313035,PMID: 25542975,PMID: 19374142
ruxolitinib	MARK2	PMID: 32353859
midostaurin	MARK2	PMID: 32353859
oseltamivir	NEU1	DOI: https://doi.org/10.1016/S2214-109X(20)30114-5
valproic acid	HDAC2	PMID: 32353859
lisinopril	ACE2	doi: 10.1001/jamacardio.2020.1624
mycophenolic acid	IMPDH2	approved drug
camostat	PRSS1	clinical trial: NCT04353284
silmitasertib	CSNK2A2	PMID: 32353859
metformin hydrochloride	NDUFAF2,NDUFAF1,NDUFB9	PMID: 32360697, PMID: 32347974
fedratinib	BRD4	PMID: 32205092
nafamostat	PRSS1	DOI: 10.1128/AAC.00754-20
gemcitabine hydrochloride	POLA1	https://www.cebm.net/COVID-19/registered-trials-and-analysis/
migalastat	GLA	PMID: 32353859
chloroquine	ACE2	PMID: 32074550
daunorubicin	ABCC1	Chemrxiv: https://orcid.org/0000-0002-4745-366X
ponatinib	RIPK1	PMID: 32353859
